# Effect of Daily Vitamin C Supplementation with or Without Flavonoids on Periodontal, Microbial, and Systemic Conditions Before and After Periodontal Therapy: A Case Series from an RCT

**DOI:** 10.3390/jcm13247571

**Published:** 2024-12-12

**Authors:** Thijs M. H. de Jong, Eleni Stamatelou, Nanning A. M. Rosema, Ineke D. C. Jansen, Bernd W. Brandt, Athanasios Angelakis, Bruno G. Loos, Ubele van der Velden, Monique M. Danser

**Affiliations:** 1 Department of Periodontology, Academic Centre for Dentistry Amsterdam (ACTA), University of Amsterdam and Vrije Universiteit Amsterdam, Gustav Mahlerlaan 3004, 1081 LA Amsterdam, The Netherlands; 2 Department of Preventive Dentistry, Academic Centre for Dentistry Amsterdam (ACTA), University of Amsterdam and Vrije Universiteit Amsterdam, Gustav Mahlerlaan 3004, 1081 LA Amsterdam, The Netherlands; 3 Department of Epidemiology and Data Science, Amsterdam University Medical Center, Location AMC, Meibergdreef 9, 1105 AZ Amsterdam, The Netherlands; 4 Digital Health and Methodology, Amsterdam Public Health Research Institute, Location AMC, Meibergdreef 9, 1105 AZ Amsterdam, The Netherlands; 5 Data Science Center, University of Amsterdam, Singel 425, 1012 WP Amsterdam, The Netherlands

**Keywords:** periodontitis, vitamin C, flavonoids, supplementation, case series

## Abstract

**Purpose:** To investigate the effect of vitamin C supplementation with or without flavonoids on periodontal conditions, and microbial and systemic variables before and after non-surgical periodontal treatment (NSPT). **Materials and Methods:** A case series derived from a randomized controlled trial was conducted to explore the effects of daily vitamin C supplementation, with or without flavonoids, on periodontal conditions. The study population was recruited from patients with periodontitis who had been referred to the Department of Periodontology at the Academic Centre for Dentistry Amsterdam (ACTA). The study consisted of a 2-month observation of untreated periodontitis followed by a 3-month period after NSPT. Descriptive statistics, correlation and clustering analyses, and dimensionality reduction methods were used to evaluate the interventions’ impact. **Results:** Due to COVID-19, the study was prematurely terminated and reported findings from 13 patients. Results indicate a correlation between higher plasma vitamin C levels and reduced gingival inflammation, suggesting benefits for untreated periodontal conditions. Clustering analysis showed no differences based on supplementation type, indicating it did not affect outcomes, and microbiological data had limited effects. Principal Component Analysis visualized clusters and illustrated no distinct groups corresponding to supplementation types. Violin plots highlighted variability, with one cluster comprising individuals with more severe periodontal conditions. **Conclusions:** Higher plasma vitamin C levels were associated with lower gingival inflammation. However, daily vitamin C supplementation, with or without flavonoids, did not show additional benefits on periodontal conditions before or after treatment. Clustering suggests that periodontal severity, rather than supplementation, influenced patient profiles. The study’s small sample size limits the generalizability of the findings.

## 1. Introduction

An important aim of the dental professional is to improve and promote periodontal health as part of oral and general health. Research over recent decades has shown that smoking, stress, and an unhealthy diet negatively affect general immune fitness, making an individual more susceptible to gingivitis and periodontitis [1,2]. Kuzmanova et al. [3] showed that periodontitis patients have lower plasma levels of vitamin C than healthy controls. In healthy controls, they also found a correlation between the level of vitamin C in the diet and the level of vitamin C in plasma. This correlation was not found in periodontitis patients. A possible explanation for the latter is that, due to a genetic variation in the vitamin C transporter protein SVCT1, periodontitis patients are less able to absorb vitamin C into their cells [4].

Vitamin C is considered an essential nutrient requiring dietary intake, as humans lack the capacity to synthesize it [5]. Severe vitamin C deficiency is associated with scurvy-related periodontitis [6] and necrotizing periodontitis [7]. There are two main reasons for this. First, functioning as a potent antioxidant, vitamin C plays a crucial role in reducing oxidative stress [8]. Second, it is integral to various physiological processes, particularly collagen synthesis, acting as a specific electron donor for enzymes involved in collagen hydroxylation [9]. Furthermore, neutrophils can regulate ascorbate uptake from plasma to maintain optimal intracellular levels for proper function. Increased oral consumption can have a positive effect on neutrophil function [10] and may contribute to reducing tissue damage and inflammatory pathologies linked to NET formation [11]. Especially in patients with conditions such as metabolic disorders or cardiovascular disease, it has the potential to reduce the risk of infection, even for COVID-19 [10].

A study on the natural history of periodontitis in Indonesia found that the severity of periodontitis was negatively correlated with the consumption of guava fruit [12], which is high in vitamin C [13]. Supplementation of vitamin C and flavonoids in this population showed a positive effect on systemic health, as indicated by reduced levels of HbA1c and hs-CRP in all subjects. In a study on the effects of kiwi consumption on periodontal and systemic health before and after initial periodontal treatment, Graziani et al. [14] found reduced gingival bleeding scores with daily kiwi consumption in a pre-treatment phase without any periodontal instrumentation or instructed changes in patient’s behavior.

Of all fruits, guava contains the highest concentration of vitamin C (228 mg/100 g) [15]. It also contains flavonoids [16]. Flavonoids, such as quercetin, have several characteristics that could also explain any possible positive influences on periodontal conditions. Quercetin is a polyphenolic flavonoid found in plant-based foods such as onions, citrus fruits, apples, red wine, berries, red grapes, and tea. Its properties have positive effects on the immune system, such as antioxidant and anti-inflammatory effects, by reducing inflammatory biomarkers, including CRP and IL-6 [17], and it may also improve endothelial function [18]. In summary, sufficient essential nutrient intake can be an important factor for proper immune fitness.

The basic and initial part of therapy in periodontal patients is non-surgical periodontal treatment (NSPT), which consists of supragingival and subgingival plaque and calculus removal, as well as thorough oral hygiene instructions. NSPT aims to reduce periodontal inflammation, arrest disease progression, and reduce pocket depth, preferably by achieving complete pocket closure. This process of healing inflamed periodontal tissues can also be considered to be a form of wound healing, as NSPT aims to convert ulcerated pocket epithelium into noninflamed epithelium attached to the root surface and to replace granulation tissue with healthy gingival connective tissue.

For complete wound healing and resistance to renewed progression of periodontitis, another important aim of the treatment is to improve host resistance. In this respect, smoking cessation and stress reduction have been studied extensively [19]. A healthy diet consisting of fruits and vegetables also contributes to improved host resistance and proper immune fitness [20,21]. Standard periodontal treatment may, therefore, be supported by using adjunctive nutritional supplements. In general, there is growing interest in adding vitamin C and flavonoid supplements to the treatment of chronic diseases [22,23]. As periodontitis is a chronic disease related to a dysbiotic microbiome in the periodontal pockets, the finding that quercetin also exhibits anti-bacterial capacities in vitro is of considerable interest [24,25].

Based on the above, it may be suggested that vitamin C supplementation, alone or in combination with flavonoids, could improve host immune fitness and, thereby, the periodontal condition and, subsequently the results of periodontal therapy. Therefore, we aimed to investigate the effect of vitamin C supplementation, with or without flavonoids, on periodontal conditions and microbial and systemic variables: (1) over a 2-month period of untreated periodontitis and (2) over a 3-month period after NSPT. It was hypothesized that the periodontal condition in untreated periodontitis patients could be improved through vitamin C supplementation alone and even more so with the addition of flavonoids, both during the period between intake and the start of the NSPT and 3 months after NSPT. To our best knowledge, this is the first time that daily vitamin C supplementation, with or without flavonoids, has been investigated in untreated periodontitis. This study was initiated as a three-arm, randomized, controlled clinical study. However, due to the COVID-19 epidemic and restricted access to the dental clinic for research patients, the study had to be terminated prematurely. Here, we report on the patients who had been enrolled.

## 2. Material and Methods

### 2.1. Study Design and Study Population

**Figure 1:**
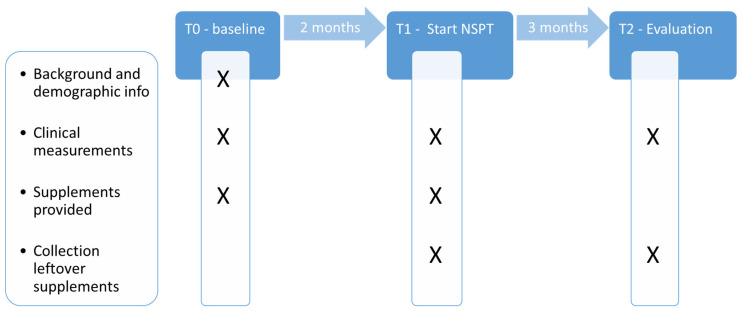
Timeline and procedures performed.

The study was designed as a single-blind, three-arm-parallel, randomized, controlled, longitudinal clinical trial with a total duration of five months (Figure 1). These five months consisted of two parts: part 1 (T0–T1) was an observational period of two months between intake and the start of the actual clinical treatment; part 2 (T1–T2) was a 3-month period between the start of the active periodontal treatment and re-evaluation. Three study groups were designed, each of which received different supplementation: (1) vitamin C with quercetin and other flavonoids (test group 1); (2) vitamin C alone (test group 2); or (3) fibers (control group); see further below under “investigational product and treatment”.

The calculation of the sample size necessary for this study (75 participants, with 25 per arm at the end of recruitment) was based on a previous study by Staudte et al. [26], which found a reduction in bleeding scores from 1.68 ± 0.6 to 1.05 ± 0.6 after grapefruit consumption. We used the bleeding scores as the primary outcome variable, assuming a standard deviation of 0.6. We concluded that 25 participants would be required per treatment arm to provide the 95% power needed to detect a difference of 0.63 between the two test groups and a control group. To compensate for possible dropouts, we planned to recruit a sample of 81 participants, with 27 per arm. Due to the COVID-19 epidemic and restricted access to the dental clinic for research patients, the study had to be terminated prematurely, and only a total of 13 patients were initially included.

The study population was recruited from patients with periodontitis who had been referred to the Department of Periodontology at the Academic Centre for Dentistry Amsterdam (ACTA) for periodontal diagnosis and treatment. At their first appointment, patients were examined by a periodontist for diagnostic purposes and treatment planning. If necessary, intra-oral X-ray images were taken to support these purposes. 

Inclusion criteria. To be eligible to participate in this study, a patient had to meet the following inclusion criteria: age ≥ 18 years; proximal radiographic bone loss of ≥3 mm at two non-adjacent teeth (definition of periodontitis); probing pocket depth (PPD) ≥ 3 mm; and bleeding on probing (BoP) on at least 25% of the total number of sites. Further, to be eligible, the severity of the periodontitis had to be stage III or IV, and the extent had to be generalized [27].

Exclusion criteria. The following exclusion criteria were applied: diagnosis of any acute periodontal problem that required immediate treatment; use of antibiotics within the previous 6 months; pregnancy or lactation; NSPT within the last year; current orthodontic treatment; use of any medication that may interfere with supplement ingredients or impair wound healing; current daily use of supplements containing vitamin C and/or flavonoids; previous immunotherapy (<3 months before appointment) or treatment needed for any malignancy; previous irradiation of the head and neck area; compromised immune response; inability to perform oral hygiene; Sjögren’s syndrome; the need to use antibiotics (including prophylaxis); and regular use (more than once per week) of anti-inflammatory drugs such as non-steroidal anti-inflammatory drugs (NSAIDs) or steroidal anti-inflammatory drugs (corticosteroids), since with regular use of these immunosuppressive drugs, a possible anti-inflammatory effect of vitamin C supplementation may not be measurable.

Informed consent and ethical approval. If a patient was interested in participating in this study, an information letter was provided. After a week, all patients were contacted to ask whether they still wished to participate in the project. If so, informed consent was obtained.

The current study was approved by the medical ethics committee of the VU Medical Centre, Vrije Universiteit Amsterdam (registration number: 2018.039); it was registered in a clinical trial database (Dutch Trial Register, clinicaltrialregister.nl, NL63480.029.18).

Randomization. The patients were randomly assigned to one of the two test groups or the control group. Randomization was performed by a computer-generated sequence with stratification for sex (female vs. male), smoking habits (current smoker vs. former or non-smoker), and overweight (Body Mass Index ≤ 25 kg/m^2^ vs. >25 kg/m^2^), giving eight options in total. Eight sets of numbers were obtained for each option. A range from one to three was used, which corresponded to one of the two test groups or the control group. To determine to which of the eight options the patient belonged, the researcher who distributed the supplements extracted the necessary information from the patient file. Subsequently, the list with eight groups of computer-generated sequence numbers was used to allocate the patient to test group 1, test group 2, or the control group.

### 2.2. Clinical and Microbiological Assessments

A calibrated periodontist (MD) obtained the following information, parameters, and samples through interviews, physical measurements, or intraoral measurements at baseline (T0), T1, and T2 (Figure 1):

At baseline (T0):

- Background and demographic information: age, sex, ethnicity, education.
- Smoking habits: tobacco smokers are defined as individuals who have smoked >1 cigarette, pipe, or cigar per day. The smoking habit was quantified by calculating pack-years (the number of packs smoked per day multiplied by the number of years smoking, assuming 20 cigs./pack). Non-smokers were individuals who had never smoked in the last 10 years or had smoked less than one pack per year in their lifetime; former smokers were individuals who had stopped smoking at least 6 months before entering the study but had not quit for more than 10 years (modification of Kuzmanova et al., 2012 [3]).

At all time points (T0, T1 and T2):

- Dietary analysis: patients were questioned about fruit, vegetable, and wine consumption in the last seven days and on the previous day. They were asked the following questions:
  - What type of fruit have you eaten in the last seven days? How many units?
  - What type of fruit did you eat yesterday? How many units?
  - Which vegetables have you eaten in the last seven days? How many multiples of 100 g?
  - Which vegetables did you eat yesterday? How many multiples of 100 g?
  - What type of wine have you drunk in the last seven days (red/white)? Number of units?
  - What type of wine did you drink yesterday (red/white)? Number of units?
- Anthropometrics: the following physical measurements were taken:
  - Weight: in kilograms (kg)
  - Length: in centimeters (cm).
  - Body Mass Index (BMI): the weight in kilograms (kg) divided by the square of the height in meters (m).
  - Waist circumference: in centimeters (cm) measured at the level of the umbilicus.
- Blood pressure (BP): systolic and diastolic blood pressure with an electronic device (OMRON M6, HEM-7001-E, OMRON HEALTHCARE Co., Ltd., Kyoto, Japan) on the right arm (brachial artery) was measured three times consecutively in a sitting position; the average of the second and third measurements was used.
- Periodontal parameters: full mouth PPD, BoP, recessions (REC; positive and negative), and plaque at six sites per tooth. Clinical attachment levels (CAL) were calculated based on PPD and REC. Periodontal inflamed surface area (PISA) was determined and calculated using PPD, BoP, and CAL [28].
- Microbiological analysis: the deepest pocket in each quadrant was selected for microbiological sampling, resulting in a total of four sampled pockets per patient. The same sites were sampled at each follow-up visit for these analyses. After each site had been isolated with cotton rolls, the supragingival plaque was removed with a Gracey curette, and the site was dried with gentle airflow. Subsequently, two medium-sized paper points (Henry Schein, Almere, the Netherlands) were inserted into the pocket for 10 s. All paper points were pooled, transferred to a sterile tube, and stored at −80 °C. At the end of the study, all samples were thawed for microbiological analysis by 16S rRNA gene amplicon sequencing. Amplicons of the V4 region were sequenced on an Illumina MiSeq, after which the paired-end sequencing data were processed as described previously [29].

### 2.3. Blood Collection and Analysis

At T0, T1, and T2 overnight fasting blood samples were obtained between 8–11 a.m. by venipuncture in the antecubital fossa. The venous blood was collected in three vacuum tubes. One K2E (EDTA) tube of 4 mL (BD-Plymouth PL6 7BP, UK), one heparin (LH PST II) tube of 3 mL (BD-Plymouth PL6 7BP, UK), and one heparin (LH Lithium Heparin) tube of 4 mL (Vacuette, Greiner Bio-One GmbH, Kremsmünster, Austria).

The K2E (EDTA) tube of 4 mL and the heparin (LH PST II) tube of 3 mL were left at room temperature and transported within 2 h to the certified biochemical laboratory of the VU University Medical Center for routine diagnostic measurements. The following biochemical measurements were performed: HbA1c, high-density lipoprotein, low-density lipoprotein, total cholesterol, triglycerides, and creatinine.

The heparin (LH Lithium Heparin) tube of 4 mL was used for plasma vitamin C and high-sensitivity C-reactive protein (hs-CRP) analysis in our laboratory. Plasma was obtained by centrifugation with an Eppendorf centrifuge type 5804R (Eppendorf AG, Hamburg, Germany) at 2000× *g* at 4 °C for 10 min. Thereafter, aliquots were made. For vitamin C, an internal standard (Chromsystems Instruments & Chemicals GmbH, Munich, Germany) was added to the samples. Plasma was stored at −80 °C. At the end of the study, all samples were thawed, and vitamin C analysis was done according to the protocol (Chromsystems Instruments & Chemicals GmbH, Vitamin C Diagnostics Kit by HPLC, Munich, Germany) as described previously [3]. For hs-CRP analyses, a hs-CRP ELISA Kitt (Inc., San Diego, CA, USA) was used according to the protocol. The sensitivity of the hs-CRP ELISA Kit was 0.1 mg/L.

### 2.4. Investigational Product and Treatment

The intervention was the intake of 1×/day of food supplements in the two test groups or the intake of non-active supplements in the control group. For part 1 (T0-T1), the participants in group 1 were provided with supplements containing vitamin C with quercetin and other flavonoids (quercetine complexe Ester-C, with vitamin C in the form of 500 mg of calcium-L-ascorbaat (Ester-C); Solgar Vitamins (Holland) B.V., Heiloo, The Netherlands), group 2 was given supplements with vitamin C alone (Ester-C 500 mg; Healthy Vitamins B.V., Zaandam, The Netherlands), whereas the control group was provided with fiber supplements (Psyllium husk; Healthy Vitamins B.V.). All three groups did not receive any periodontal treatment in part 1; two months is the typical duration of ‘waiting time’ between intake and the first actual treatment appointment. At the start of part 2, all groups received full mouth NSPT, which was performed in two appointments of 2.5 h each within 2 weeks, and oral hygiene reinforcement after 6 weeks. The NSPT was performed at the Department of Periodontology, ACTA, by two selected experienced dental hygienists.

In part 2 of the study, all groups were instructed to continue taking the assigned supplements. All patients had to keep a record of which they had to fill in per day, and whether they had taken their supplements, a missed supplement was not to be compensated. At the recall appointment, leftover supplements were returned and counted. The periodontist who performed all the measurements, as well as the dental hygienists who performed the periodontal treatment, were blinded to the type of supplements. However, the patients were not blinded but were requested to refrain from sharing experiences. All products were provided free of charge during the course of the study.

Adverse events, defined as any undesirable experience occurring in a subject during the study, whether or not considered related to the intervention, were reported spontaneously by the subject or observed by the staff and were recorded.

### 2.5. Data Analysis

Two types of data analysis were performed: descriptive statistics and cluster analysis.

Descriptive statistics. Statistical analyses (median and Spearman’s correlation analysis) were performed with IBM SPSS for Windows (Version 28.0, Released 2021, IBM Corp., Armonk, NY, USA), and graphs were generated with GraphPad software (GraphPad Prism Version 8.1.0, San Diego, CA, USA).

The following variables were included: background and demographic information (age, sex, and education) and smoking habits (smoker and pack years). For each time point, we included the anthropometrics (BMI and WC), the BP measurements, the fruit, vegetables, and wine intake over the last seven days and the previous day, the blood test results (hs-CRP, HbA1c, high-density lipoprotein, low-density lipoprotein, total cholesterol, triglycerides, creatinine, vitamin C), the periodontal measurements excluding third molars (PISA, BoP, mean PPD [both all sites and interproximal], mean CAL interproximal), and 32 microbiological genera.

Cluster analysis. Clustering was performed with Python (v.3.9.6.), including all variables listed above, to explore whether the type of supplementation affected patient periodontal profiles. For this approach, patient 6 was excluded due to missing data at the time point T2; the limited number of participants prohibited us from applying any state-of-the-art data imputation technique. To normalize the data, we applied min–max normalization. Using the k-means algorithm, known for its soundness and robustness [30,31,32], we chose two clusters to investigate the potential impact of vitamin C supplementation versus fibers. We set the ‘random_state’ parameter to 42 for reproducibility, ‘n_clusters’ to 2, and for the other parameters, we used the default values.

We conducted two cluster analyses; first, we included all variables without the microbiological data, and thereafter, we included the same variables plus the microbiological data. For each clustering, we considered three different cases, including each data from different time points: T0; T0 and T1; T1 and T2. At T0, we used the variables of T0 to cluster the subjects at baseline to ascertain that subjects assigned to vitamin C and controls were randomly assigned into two groups with the same ratio of assignment. After two months of supplementation before the periodontal therapy, we applied clustering using the variables at T0 (to account for baseline variables) and T1 to investigate whether the ratio of assignment changed. After the periodontal treatment, we clustered the subjects using the variables from T1 (to account for the type of supplementation given in the preceding two-month period) and T2.

Visualization methods. For dimensionality reduction, we applied three-dimensional Principal Component Analysis [33,34] (3D PCA) in Python (v.3.9.6.). The 3D PCA, which reduces the features’ space linearly, provided the most informative way to visualize the two clusters from each cluster analysis. To enhance the visual distinction between the clusters, we introduced a two-dimensional plane within the 3D PCA space. This plane, derived from the separation boundary of the Support Vector Machines algorithm [35], was calculated to maximize the distance between the plane and the nearest points from each cluster. For illustrative purposes, we presented a two-dimensional projection of the 3D PCA that best demonstrates the distinct separation of the two clusters. In this separation, the 2D plane appears as a line, offering a clear visual representation of the division between the clusters.

Finally, violin plots were generated with Python (v.3.9.6.) to investigate and visualize the differentiating characteristics of each cluster. These plots are particularly informative, combining the information from a box plot and a kernel density plot to provide a summary of the data.

## 3. Results

Descriptive statistics. Thirteen patients consented to participate in the case series, eleven subjects completed period 1, and ten subjects also completed period 2 (Supplementary Figure S1). Table 1 presents the background characteristics of the participants in consecutive order of enrollment. There were four females and nine males, with an age range of 29 to 65 years. Six subjects were smokers, four were former smokers, and three were non-smokers. Four participants had a BMI < 25 kg/m^2^, three subjects had a BMI between 25 and 30 kg/m^2^, and six showed to have a BMI > 30 kg/m^2^, of which subject 7 even had a BMI of around 40 kg/m^2^. Eleven subjects had an education level of at least high school or beyond. One of the patients, subject 10, had type 2 diabetes mellitus and used medication (Metformine and Gliclazide). Subjects 1 and 4 had high blood pressure and were taking medication for this. Four patients, subjects 1, 7, 10, and 12, were taking cholesterol-lowering agents, and three patients, subjects 2, 6, and 13, were taking other medication (platelet aggregation inhibitor, painkiller, and antiviral agent, respectively).

**Figure 2:**
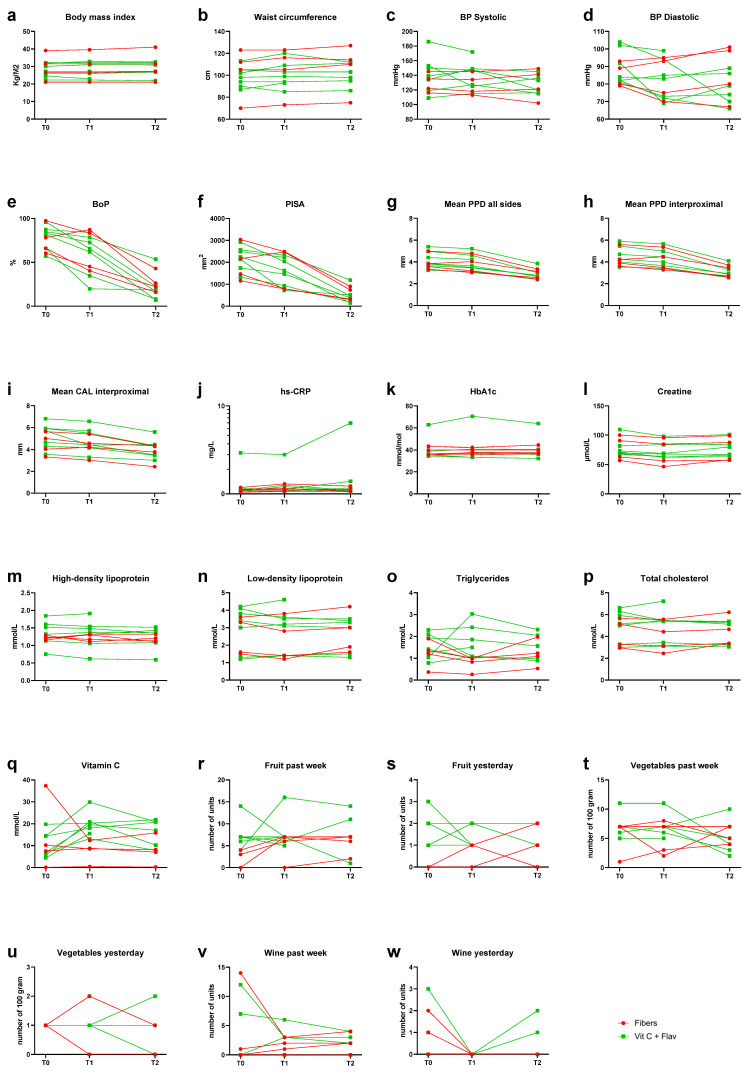
Interleaved scatter plots of parameters at T0, T1, and T2. (**a**): Body mass index (BMI). (**b**): Waist circumference (WC). (**c**,**d**): Blood pressure (BP) systolic (**c**) and diastolic (**d**). (**e**): Bleeding on probing (BoP). (**f**): Periodontal inflamed surface area (PISA). (**g**,**h**): Mean probing pocket depth (PPD) on all sides (**g**) and interproximal (**h**). (**i**): Clinical attachment level (CAL). (**j**): High sensitivity C-reactive protein (hs-CRP). (**k**): HbA1c. (**l**): Creatine. (**m**): High-density lipoprotein. (**n**): Low-density lipoprotein. (**o**): Triglycerides. (**p**): Total cholesterol. (**q**): Vitamin C levels. (**r**–**w**): Consumption of fruit (**r**,**s**), vegetables (**t**,**u**), and wine (**v**,**w**) in the last seven days (last week) and on the previous day (yesterday).

The supplementation allocation based on randomization is also presented in Table 1. Six periodontitis patients were randomized to receive vitamin C with flavonoids, two individuals to vitamin C alone, and the remainder to fibers. Two subjects missed more than two days of taking the supplements. One adverse event occurred during the study and was related to the intervention. A patient in the fiber group, subject 8, suffered from constipation and, therefore did not always take the supplements. Subject 13 forgot to take the supplements more than twice. For a description of the results, we combined test group 1 (vitamin C with flavonoids, *n* = 5) with test group 2 (vitamin C alone, *n* = 2). Figure 2a–d show parameters BMI, WC, and BP at T0, T1, and T2. For BMI and WC, values remained comparable to baseline throughout the observation period, irrespective of the supplementation.

In Figure 2e,f, we observe that three out of four cases of the fiber group and all seven cases of the vitamin C group showed some reduction in BoP and PISA between T0 and T1. At baseline, the BoP ranged from 57% to 97%, and the PISA ranged from 1161 mm^2^ to 3038 mm^2^. At T1, the BoP and PISA were reduced for ten out of eleven subjects, seven in the vitamin C group and three from the fiber group. From T1 to T2, a reduction in BoP and PISA was seen in all patients. The BoP ranged from 7% to 54%, and the PISA ranged from 147 to 1191 mm^2^. Five subjects showed at T2 a BoP below 20%, four out of six from the vitamin C and flavonoids group and one out of four from the fiber group.

Figure 2g–i show the results for PPD and CAL. The mean PPD at T0 ranged from 3.25 to 5.40 mm. At T1, the PPD ranged from 3.03 to 5.22 mm^2^ and was reduced for ten out of eleven subjects, seven vitamin C patients, and three from the fiber group. From T1 to T2, we noted substantial PPD reductions ranging from 2.43 to 3.85 mm^2^. Six subjects showed at least 1 mm mean PPD reduction: four out of six from the vitamin C and flavonoids group and two out of four from the fiber group. One patient of the fiber group, subject 1, showed an increase in BoP, PISA, mean PPD, and mean CAL between T0 and T1. One patient of the vitamin C group, subject 13, showed only a reduction in the mean PPD for all sides but not for the interproximal sites. Interestingly, the mean CAL interproximal in those subjects who received vitamin C was more pronounced from T0 to T1 (the period with supplementation alone) than in the subjects with fibers. Between T1 and T2, after active non-surgical periodontal treatment, all subjects showed a substantial reduction in BoP, PISA, mean PPD, and CAL.

Figure 2j–p present the biochemical parameters. Subject 9 was the only patient with hs-CRP > 3 mg/L at baseline. Besides periodontal disease, this patient was medically healthy. Subject 10 had elevated (>53 mmol/mol) HbA1c levels; this was a patient with type 2 diabetes. Seven patients had elevated (≥5.0 mmol/L) total cholesterol: two in the fiber group (subjects 4 and 7) and five in the vitamin C and flavonoids group (subjects 2, 6, 9, 11, and 13). Of these subjects, at intake, only subject 7 was known to have elevated cholesterol and used medication for this. Subjects 1, 10, and 12 also reported at intake that they had elevated cholesterol and were taking medication for this. Most of the subjects with elevated cholesterol showed some reduction between T0 and T1 and between T1 and T2. Only subject 4 showed an increase in both time periods, from 3.60 mmol/L at T0 to 3.80 mmol/L at T1 and 4.20 mmol/L at T2, whereas low-density lipoprotein ≤ 2.5 mmol/L is seen as optimal and ≥3.5 mmol/L is too high.

**Figure 3:**
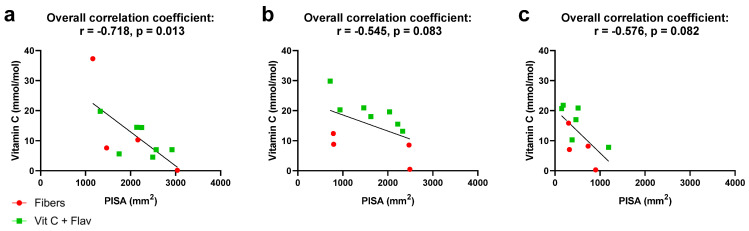
Spearman correlation between PISA and vitamin C at T0 (**a**), T1 (**b**), and T2 (**c**).

Plasma vitamin C levels at T0 ranged from 0.15 to 37.32 mg/L (Figure 2q). In the subjects who were assigned to take a daily dose of supplements with vitamin C, we noted a clear increase. At baseline, four out of seven subjects had plasma levels between 4 and 10 mg/L, and at T1, all subjects in the vitamin C and flavonoids group had levels above 10 mg/L. For six out of seven subjects, this was still the case at T2. For the participants in the fiber group, no increase in plasma vitamin C was seen from T0 to T1 and from T1 to T2. One of them, subject 7, showed vitamin C deficiency at all time points. To ensure that increases in vitamin C were indeed related to supplementation and to interpret the individual results for other subjects, we questioned the consumption of fruit, vegetables, and wine (Figure 2r–w). Coincidentally, those who took vitamin C and flavonoids showed a higher intake of fruits at baseline and during the study. In fiber group 2, subjects had not eaten fruit for a week at T0, and the other two subjects had less than one piece on average per day. In the vitamin C and flavonoids group, five out of seven subjects had on average at least one piece of fruit per day. When we explored the relationship between PISA and vitamin C levels for all participants, it was seen that at T0, PISA correlated with vitamin C levels (r = −0.718, *p* = 0.013) (Figure 3). These correlations were not observed at T1 and T2. Interestingly, subject 7, being extremely obese and vitamin C deficient at all time points, showed improvement in BoP, PISA, PPD, and CAL at T1 and, more clearly, at T2.

**Figure 4:**
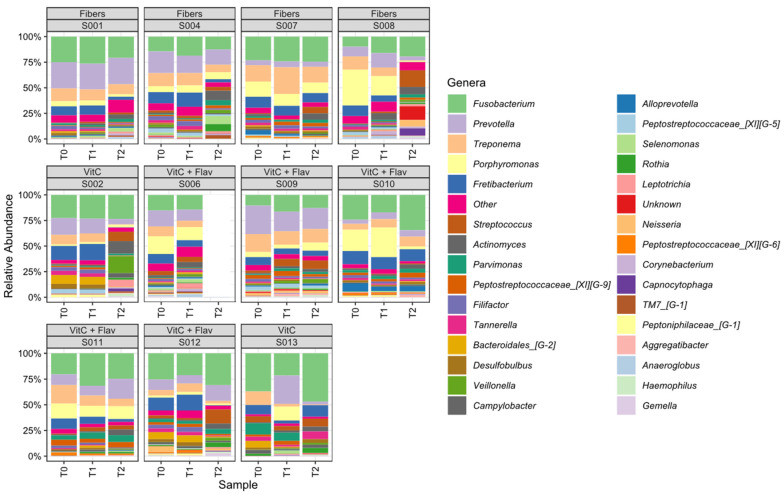
Relative abundances of genera are shown per individual. “Unknown” refers to taxa unclassified at the genus level, while “Other” combines genera present at a relative abundance of less than 2% in all samples.

Figure 4 shows a genus-level summary of the different bacteria present per subject. *Fusobacterium* was the most observed genus, followed by *Prevotella*, *Treponema*, *Porphyromonas*, and *Fretibacterium*. The relative abundance of *Fusobacterium* at T0 ranged from 15% to 45%. The relative abundance of *Prevotella*, *Treponema*, and *Porphyromonas* at T0 ranged from 0% to 26%, from 5 to 18%, and from 0% to 36%, respectively. The relative prevalence of these four genera summed to more than 50% in ten out of eleven patients at baseline. At T1, a reduction in the relative abundance of these four genera was seen in seven out of eleven subjects, five out of seven from the vitamin C and flavonoids group, and two out of four of the fiber group. At T2, in all four subjects of the fiber group, a reduction in the relative abundance of these four genera was seen compared to T1; for the vitamin C group, this was only the case for one patient, subject 2.

The combined five asaccharolytic, anaerobic genera *Fusobacterium*, *Prevotella*, *Treponema*, *Porphyromonas*, and *Fretibacterium* occurred at baseline (T0) in the range of 56% to 77% for all subjects, while 3 months after non-surgical periodontal therapy (T2), their relative prevalence was generally lower, now ranging from 25–67%. At T1, there were only minor changes, but at T2, in all patients, *Streptococcus* and *Actinomyces* showed a higher relative abundance compared to T1, combined ranging from 0–6% at T0 to 5–23% at T2. A clear difference between patients who took vitamin C supplements, with or without flavonoids or fibers, was not visible.

**Figure 5:**
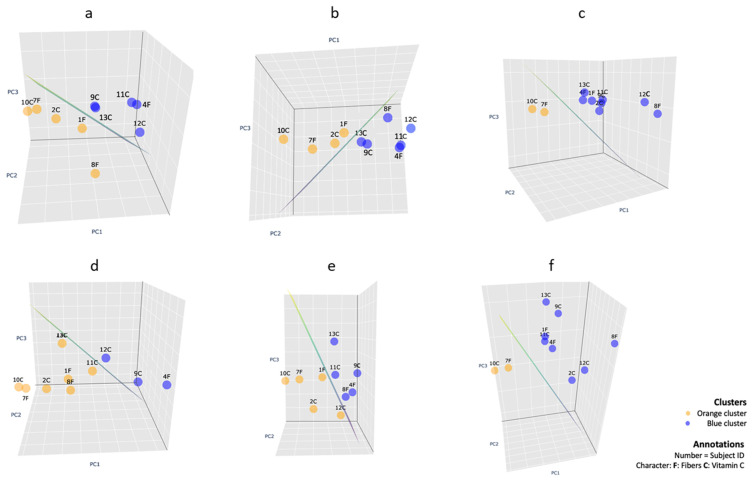
This figure presents three-dimensional Principal Component Analysis (PCA) visualizations of individuals classified into two clusters (represented in orange and blue) based on k-means clustering results. The plane that separates the two clusters is visible as a green line from the selected perspectives. The annotations at each data point consist of the unique Subject ID followed by a letter indicating the supplement group: ‘F’ for Fibers and ‘C’ for vitamin C. Panels a to c illustrate the PCA outcomes at time points T0 (**a**), combined T1 and T0 (**b**), and combined T2 and T1 (**c**), without including microbiological data. Panels (**d**–**f**) represent the PCA with the same variables as in panels a to c but include microbiological data (32 genera at each time point).

**Figure 6:**
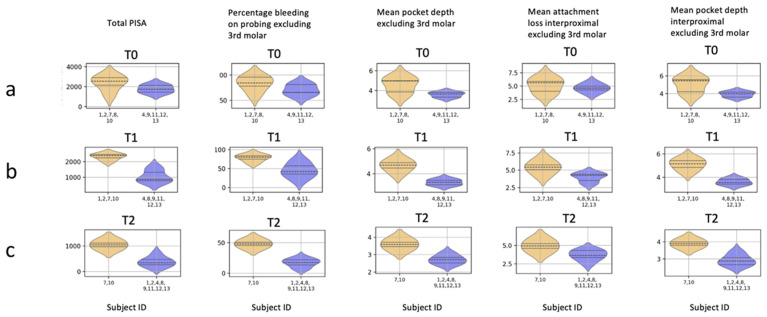
This figure presents violin plots of periodontal variables for two clusters (depicted in orange and blue) derived from k-means clustering without the microbiological data presented in Figure 5a–c. Each violin plot displays the median using a dashed line, the interquartile range using dotted lines, and a rotated kernel density plot, which shows the data distribution for that variable. These selected periodontal parameters differentiated the clusters the most. The violin plots show the periodontal variables from the clustering at T0 (Row (**a**)), at T1 from clustering T0 and T1 (Row (**b**)), and at T2 from clustering T1 and T2 (Row (**c**)).

Cluster analysis and visualization. To explore patient profiles while including the selected variables simultaneously at baseline, after supplementation, and after NSPT, we performed cluster analysis. In Figure 5, we selected the angle where the two clusters of the 3D plot were linearly separated without the microbiological data (Figure 5a–c) and with the microbiological data (Figure 5d–f). Each subject’s cluster data point across different cases is presented with annotations indicating the Subject ID and the supplementation group type (‘F’ for fibers and ‘C’ for vitamin C), allowing for an examination of each subject trajectory at different time points in the study. At baseline T0 (Figure 5a), we observed two heterogeneous clusters, indicating that individuals were indeed randomly allocated into the fibers or vitamin C supplementation group; the individuals in the two clusters differed mainly in the severity of periodontitis, as can be seen in the violin plots in Figure 6a (PISA, BoP, mean PPD, mean AL interproximal, mean PPD interproximal). At clustering of combined T0 and T1 data (Figure 5b), we noted that all the individual trajectories, except for subject 8 (Fiber), remained in the same cluster as in the T0 clustering, based on their severity of periodontitis (Figure 6b), indicating essentially no effect of vitamin C supplementation in the pre-treatment phase. At combined T1 and T2 clustering (Figure 5c), we noted that subjects 7 (fiber) and 10 (vitamin C) remained in the orange cluster, while subjects 1 (fiber) and 2 (vitamin C) were clustered in the blue cluster. The remaining subjects were in the blue cluster, showing that the values of the periodontal variables were lower in the blue cluster (Figure 6c), suggesting that the type of supplementation did not affect the outcome of NSPT. When we repeated the cluster analysis while including the microbiological data (Figure 5d–f), the effect of the microbiological data on the clustering was limited. At T0 clustering (Figure 5d), two additional subjects, 11 (vitamin C) and 13 (vitamin C), were clustered in the orange cluster compared to Figure 5a. At combined T0 and T1 clustering, subject 12 (vitamin C) was clustered differently into the orange cluster, compared to Figure 5b. At combined T1 and T2, all the subjects were clustered in the same clusters as in Figure 5c. Further analysis of the two clusters at the three time points showed, again, the orange cluster exhibiting higher values for periodontal variables than the blue cluster.

## 4. Discussion

In the current study, we investigated the effect of vitamin C supplementation, with or without flavonoids, on the periodontal conditions and on microbiological and systemic variables; first, after a 2-month “waiting” period before treatment of periodontitis, and then a 3-month period after NSPT. The strength of this study was that it is the first time that daily vitamin C supplementation, with or without flavonoids, has been investigated in untreated periodontitis. The vitamin C supplements, with or without flavonoids, were well tolerated. One patient in the fiber group suffered from constipation. With regard to Psyllium Husk fibers, the dose of 500 mg used in this study was much lower compared to the dose used in a safety study on psyllium fibers [36]. The originally planned RCT was abandoned due to the COVID-19 crisis, and the recruitment of patients was discontinued. Before the pandemic, we included 13 periodontitis patients randomly in the study; here, we report on these cases. Within the limitations of the current study, including only 13 cases, the periodontal inflammation (BoP and PISA) was reduced to a small extent in the pre-treatment phase, regardless of the type of supplementation. This trend was not seen for PPD and CAL. The limited number of patients prohibited us from performing statistical analyses on ‘group’ differences. Nevertheless, we noticed that the level of inflammation at baseline correlated with lower levels of vitamin C, and this trend was also observed in the “waiting” period before treatment and after NSPT. This corroborates with previous findings [3,37,38,39]. When we evaluated the clinical periodontal results after NSPT, we noted a substantial reduction in the periodontal variables (BoP, PISA, PPD, and CAL), irrespective of the supplementation type and comparable to a previous investigation in our clinic [40]. Furthermore, the anthropometric variables, blood pressure measurements, biochemical results, fruit, vegetable, and wine consumption, and microbiological variables did not indicate any specific effect of vitamin C supplementation. To explore patterns in patient profiles while including multiple variables simultaneously, we applied cluster analysis. The clusters demonstrated a mix of subjects with either fiber or vitamin C supplementation at different time points, again indicating that vitamin C did not have a notable influence compared to the impact of the pre-treatment or post-treatment periodontal variables. The consistency of individuals remaining within the same cluster across different time points, irrespective of the type of supplementation, was not explained by the vitamin C supplementation but rather by the variation of the underlying periodontal severity. When we repeated the clustering analysis at different time points, including microbiological variables, most of the individuals remained clustered in the same clusters. Such consistency underlines the robustness of the clustering against the variability introduced by the microbiological data, suggesting that for forming the clusters, the individual severeness of the periodontal condition based on PISA, BoP, PPD, and CAL was dominantly deterministic over the microbiological data, as seen in Figure 5 and Figure 6.

In this study, it was hypothesized that the periodontal condition in untreated periodontitis patients can be improved through vitamin C supplementation, both during the period between intake and the start of the NSPT and 3 months after NSPT. There is no literature about the direct relationship between vitamin C levels and PISA in patients with periodontal disease. In the current case series, we did find a negative correlation between vitamin C and PISA at baseline and similar trends after the “waiting” period and after NSPT. There are several studies indicating the association between vitamin C intake or serum levels and overall periodontal health. A study involving 12,419 US adults found that a reduced dietary intake of vitamin C is associated with an increased risk for periodontal disease, with a significant effect seen in current and former smokers [41]. Another investigation, a pilot study of patients at the Westmead Centre of Oral Health Periodontic Clinic, found that six out of twenty patients had vitamin C levels less than the institutional normal range. Low vitamin C was associated with a higher periodontal disease stage [42]. In one of the studies on the Indonesian population, it was found that plasma vitamin C deficiency was associated with increased attachment loss, suggesting that vitamin C deficiency may contribute to the severity of periodontal disease [43]. It was also seen that patients with periodontitis had a significantly lower intake of vitamin C compared to healthy subjects [41,44,45]. A clinical study found a decrease in blood pressure and improvement of endothelial function by intake of polyphenolic flavonoids [46]. Quercetin-rich diets appear to reduce the risk of poor cardiovascular function [47] and reduce plasma LDL concentration levels [48]. In the current case report, we did not find any effects of vitamin C and flavonoids relating to blood pressure and cholesterol levels. In a non-clinical study, the effect of the flavonoid quercetin was found on the periodontal pathogens *Aggregatibacter actinomycetemcomitans* (*Aa*) and *Porphyromonas gingivalis* (*Pg*); when evaluating the effect on the growth of *Aa* and *Pg*, a significant decrease in viable counts after 1 h was observed when quercetin solution was added to suspensions of these bacteria [25]. The results suggest that quercetin possesses significant antimicrobial properties on periodontal pathogens in vitro and, as such, could have an effect on the pocket ecosystem. This in vitro observation was reflected in the relative abundance of the genera in our study.

The current report has several limitations. The first and major limitation is that only 13 patients with periodontitis were included due to the COVID-19 pandemic. Only necessary patient treatments were allowed, while the clinics of ACTA stopped clinical research. Due to the limited sample, we were not able to apply comparative statistics between the groups. The only statistical test we could perform was the non-parametric correlation test between vitamin C levels and PISA, irrespective of group allocation. Next to that, we presented the descriptive statistics for all variables for future reference. However, to extend the explorative analysis of the study, we used cluster analysis to discover trajectories in patient profiles while including multiple variables simultaneously, adjusting for baseline variables with or without microbiological variables. The patterns in the patient profiles had similarities in the periodontal variables, and the impact of vitamin C was not notable. The clustering with the microbiological variables minimally affected those patterns. The microbiological profiles reflect the periodontal severity that is already reflected by the clinical periodontal variables, indicating that the microbiological profiles follow clinical severity. These results confirm the lack of the effect of vitamin C observed on individual variables in the current study. A second limitation could be that the daily dose of 500 mg of vitamin C in this study was too low. The choice of the dose was based on the most common dose sold over-the-counter and, therefore, consumed in the Netherlands. A higher dose of vitamin C supplementation may have yielded a more substantial impact of this adjunctive intervention. Research indicates that effective doses of vitamin C supplements for improving diabetes outcomes and lowering the risk for cardiovascular disease range from 500 to 1000 mg per day [49,50]. Most individuals can safely take up to 1000 mg daily, but those with certain genetic conditions, like haemochromatosis or beta-thalassemia, or with severe health issues such as end-stage renal disease, should limit their intake to 500 mg per day. Nevertheless, a study by Graziani et al. [14] indicates the dosage of vitamin C in two kiwis per day (equaling approximately 100–200 mg daily) must be seen as minimal doses for patients with periodontitis. Another study by Levine et al. [51] also recommends a daily dose of at least 200 mg daily. A third limitation is that our study was designed as a single-blind, randomized, controlled clinical trial. Double-blind designs would have been preferred so that patients would also be unaware of which supplement they received. Unfortunately, it was a requirement of our medical ethical committee to leave the products in the original packaging.

## 5. Conclusions

Due to the small sample size, the current results cannot be generalized. We confirm that gingival inflammation, specifically PISA, is negatively correlated with plasma vitamin C levels. However, patients who are deficient in vitamin C can also respond positively to NSPT. The findings of the clustering analysis describe the patient profiles of our data but cannot be generalized due to the small sample size. Further RCTs with enough subjects and, therefore, sufficient power are needed to draw a definitive conclusion regarding the effects of vitamin C supplementation, with or without flavonoids, on periodontal conditions and microbial and systemic variables in patients with periodontitis. The amount of vitamin C supplementation given can be up to 1000 mg per day, but up to 500 mg is safer and most likely sufficient.

In conclusion, from the current case series with an RCT design, we corroborate that higher levels of plasma vitamin C are correlated with lower levels of gingival inflammation. However, we cannot conclude that daily intake of vitamin C supplements, with or without flavonoids, has an adjunctive impact on pre-treatment and post-treatment periodontal conditions.

## Figures and Tables

**Table 1:** Patient characteristics for the three study groups at baseline. Abbreviations: BMI, body mass index; Vit C + Flav, vitamin C and flavonoids.

Subject	Sex	Age	Smoker	Pack Years ^a^	BMI (kg/m^2^)	Education (≥High School)	Assigned to (Supplements)
1	Male	48	Yes	30.0	26.5	Yes	Fibers
2	Female	48	Former	15.0	26.0	Yes	Vitamin C
3 *	Female	52	No	-	32.8	Yes	Vit C + Flav
4	Male	60	No	-	31.5	Yes	Fibers
5 *	Male	40	Yes	25.0	24.4	Yes	Fibers
6	Male	61	Yes	30.0	24.6	Yes	Vit C + Flav
7	Female	55	Yes	30.0	39.1	Yes	Fibers
8	Female	29	No	-	21.1	Yes	Fibers
9	Male	58	Former	9.0	32.2	Yes	Vit C + Flav
10	Male	60	Yes	17.5	30.1	No	Vit C + Flav
11	Male	48	Yes	12.5	27.2	Yes	Vit C + Flav
12	Male	65	Former	68.0	22.3	Yes	Vit C + Flav
13	Male	48	Yes	30.0	31.9	Yes	Vitamin C

^a^ The number of packs of cigarettes smoked per day multiplied by the number of years smoking. * Patient retracted from study after baseline.

## Data Availability

The data used to support the findings of this study are available from the corresponding author upon request.

## Supplementary Materials

The following supporting information can be downloaded at: https://www.mdpi.com/article/10.3390/jcm13247571/s1, Figure S1: CONSORT flow chart of the study; Table S1: Body mass index (BMI), waist circumference (WC), blood pressure (BP) systolic and diastolic at T0, T1 and T2; Table S2: Periodontal clinical parameters at T0, T1, and T2: bleeding on probing (BoP), periodontal inflamed surface area (PISA), mean probing pocket depth (PPD) on all sides, interproximal and clinical attachment level (CAL); Table S3: Biochemical parameters at T0, T1, and T2: high sensitivity C-reactive protein (hs-CRP), HbA1c, creatine, high-density lipoprotein, low-density lipoprotein, triglycerides, and total cholesterol; Table S4: Vitamin C levels at T0, T1 and T2; Table S5: Consumption of fruit, vegetables, and wine at T0, T1, T2.
