# Supplementary material for: Effect of Daily Vitamin C Supplementation with or Without Flavonoids on Periodontal, Microbial, and Systemic Conditions Before and After Periodontal Therapy: A Case Series from an RCT
Source: J Clin Med. 2024 Dec 12;13(24):7571. doi: 10.3390/jcm13247571 (PMC11678909; doi:10.3390/jcm13247571)
Supplement: Supplementary file 1 [file jcm-13-07571-s001.zip › jcm-3330560-supplementary.pdf]

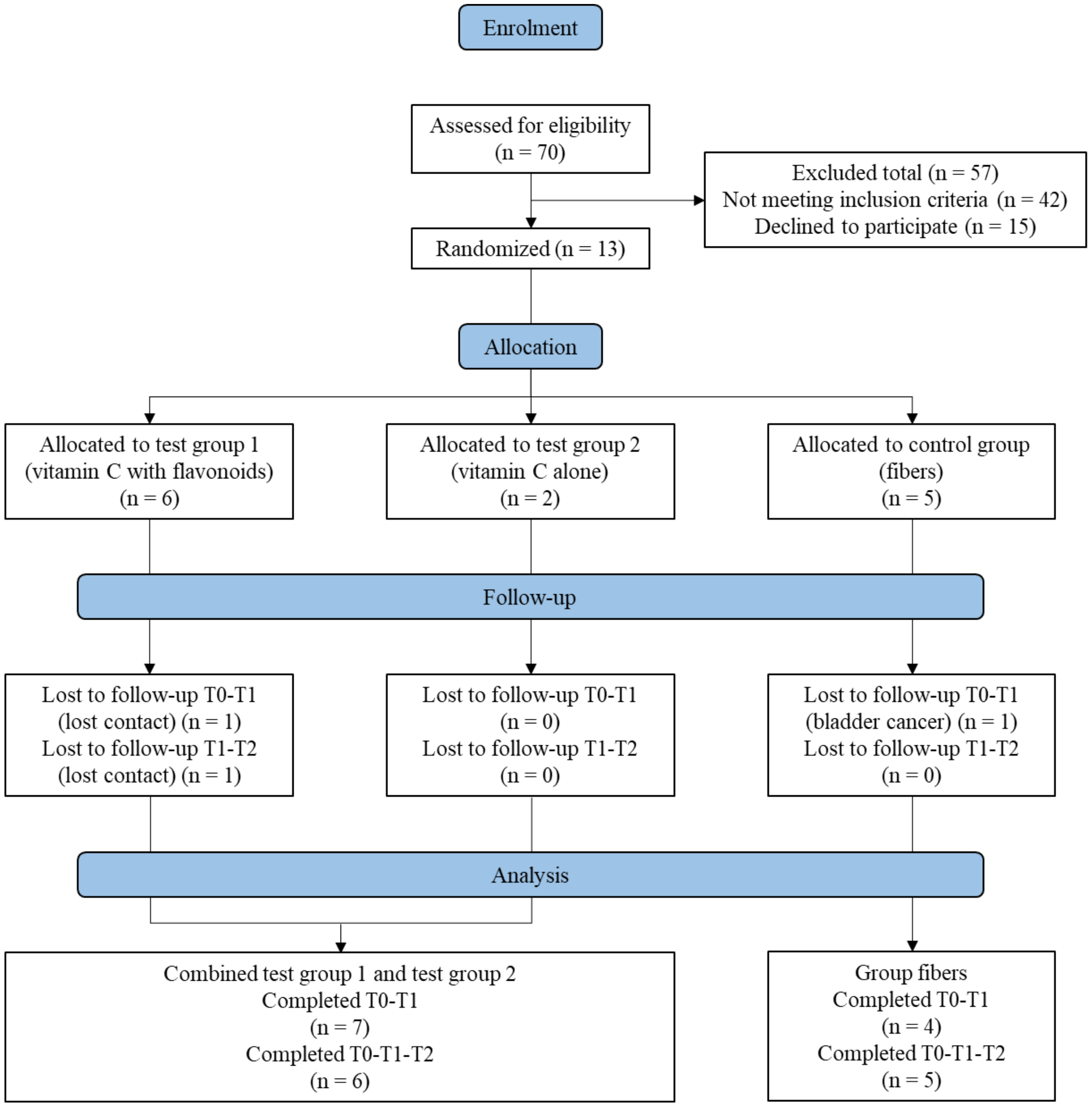

Figure S1. CONSORT flow chart of the study.

Table S1. Body mass index (BMI), waist circumference (WC), blood pressure (BP) systolic and diastolic at T0, T1 and T2.

Subject	BMI (Kg/M^2)			WC (cm)			BP Systolic (mmHg)			BP Diastolic (mmHg)		
	**T0**	**T1**	**T2**	**T0**	**T1**	**T2**	**T0**	**T1**	**T2**	**T0**	**T1**	**T2**
**Group: Fibers**												
1	26.5	26.5	27.1	105	105	110	122	118	121	80	75	80
4	31.5	31.5	31.5	112	116	114	145	145	149	93	95	99
7	39.1	39.5	41.0	123	123	127	135	134	141	89	93	101
8	21.1	21.1	21.1	70	73	75	116	113	102	79	70	67
Median	*29.0*	*29.0*	*29.3*	*109*	*111*	*112*	*128*	*126*	*131*	*84*	*84*	*89*
**Group: Vit C + Flav**												
**2**[a]	26.0	26.0	26.8	94	94	95	109	115	116	84	83	89
6	24.6	22.9	-	87	93	-	186	172	-	102	99	-
9	32.2	32.2	32.2	98	99	98	119	127	115	82	72	66
10	30.1	31.0	30.9	102	109	111	134	149	145	82	85	86
11	27.2	27.2	27.2	105	103	103	153	125	136	104	94	70
12	22.3	22.3	22.0	90	85	86	139	147	120	81	73	74
**13**[a]	31.9	32.8	32.6	113	120	112	150	147	133	92	69	79
Median	*27.2*	*27.2*	*29.0*	*98*	*99*	*101*	*139*	*147*	*127*	*84*	*83*	*76*

[a] Subject received supplements with only 500 mg of vitamin C (without flavonoids).
-, represents no data available, patient did not attend visit T2.

BP was measured 3 times consecutively; the average of the second and third measurements was used. In SPSS the unrounded values were used to calculate the median.
Abbreviations: BMI, body mass index; WC, waist circumference; blood pressure, BP.

Table S2. Periodontal clinical parameters at T0, T1, and T2: bleeding on probing (BoP), periodontal inflamed surface area (PISA), mean probing pocket depth (PPD) on all sides, interproximal and clinical attachment level (CAL).

**Subject**	**BoP (%)**			**PISA (mm^2)**			**Mean PPD (mm) all sites**			**Mean PPD (mm) interproximal**			**Mean CAL (mm) interproximal**		
	**T0**	**T1**	**T2**	**T0**	**T1**	**T2**	**T0**	**T1**	**T2**	**T0**	**T1**	**T2**	**T0**	**T1**	**T2**
**Group: Fibers**															
1	78.21	87.18	26.28	2160	2473	737	3.85	4.03	3.13	4.21	4.48	3.47	4.04	4.21	3.78
4	66.00	40.28	15.97	1467	796	322	3.34	3.03	2.56	3.61	3.28	2.71	5.00	4.55	4.33
7	97.44	83.33	42.95	3038	2491	903	4.99	4.78	3.35	5.60	5.36	3.71	5.63	5.40	4.30
8	60.42	45.14	22.22	1161	789	301	3.57	3.19	2.43	3.89	3.48	2.61	3.33	3.02	2.43
Median	*72.1*	*64.2*	*24.3*	*1813*	*1634*	*530*	*3.71*	*3.61*	*2.85*	*4.05*	*3.98*	*3.09*	*4.52*	*4.38*	*4.04*
**Group: Vit C + Flav**															
**2**[a]	95.95	65.54	16.89	2920	2038	464	4.97	4.61	3.01	5.46	4.99	3.29	5.91	5.49	4.34
6	87.33	84.00	-	2493	2221	-	4.41	4.23	-	4.70	4.46	-	5.92	5.70	-
9	83.33	72.73	22.73	1746	1467	381	3.85	3.64	2.67	4.25	3.98	2.86	4.65	4.42	3.53
10	84.72	78.47	53.62	2570	2329	1191	5.40	5.22	3.85	5.90	5.65	4.10	6.81	6.57	5.59
11	66.05	19.75	18.52	2137	718	514	3.66	3.45	2.77	4.00	3.66	2.93	5.70	4.39	4.45
12	57.33	34.67	8.00	1325	936	180	3.25	3.12	2.39	3.55	3.41	2.56	4.29	4.16	3.47
**13** [a]	81.55	61.90	7.14	2247	1623	147	3.82	3.50	2.78	4.21	4.48	3.47	3.57	3.30	3.02
Median	*83.3*	*65.5*	*17.7*	*2247*	*1623*	*381*	*3.85*	*3.64*	*2.77*	*4.25*	*3.98*	*2.97*	*5.70*	*4.42*	*3.94*

[a] Subject received supplements with only 500 mg of vitamin C (without flavonoids).

The third molars were excluded from these calculations.
-, represents no data available, patient did not attend visit T2.
Abbreviations: BoP, bleeding on probing; PISA, periodontal inflamed surface area; PPD, probing pocket depth; CAL, clinical attachment level.

Table S3. Biochemical parameters at T0, T1, and T2: high sensitivity C-reactive protein (hs-CRP), HbA1c, creatine, high-density lipoprotein, low-density lipoprotein, triglycerides, and total cholesterol.

**Subject**	**hs-CRP (mg/L)**			**HbA1c (mmol/mol)**			**Creatine (μmol/L)**			**High-density lipoprotein (mmol/L)**			**Low-density lipoprotein (mmol/L)**			**Triglycerides (mmol/L)**			**Total cholesterol (mmol/L)**		
	**T0**	**T1**	**T2**	**T0**	**T1**	**T2**	**T0**	**T1**	**T2**	**T0**	**T1**	**T2**	**T0**	**T1**	**T2**	**T0**	**T1**	**T2**	**T0**	**T1**	**T2**
**Group: Fibers**																					
1	0.44	0.41	0.53	39.9	39.9	39.9	100.1	95.5	99.0	1.15	1.32	1.32	1.60	1.40	1.60	1.20	0.83	1.08	3.27	3.12	3.37
4	0.49	0.62	0.29	35.5	37.7	37.7	90.6	84.5	87.5	1.20	1.30	1.11	3.60	3.80	4.20	1.90	0.99	1.97	5.64	5.56	6.21
7	0.19	0.28	0.30	43.2	42.1	44.3	63.0	56.3	57.3	1.24	1.17	1.13	3.30	2.80	3.00	1.35	1.00	1.23	5.12	4.43	4.65
8	0.72	1.13	0.88	35.5	35.5	36.6	57.1	46.5	58.2	1.29	1.11	1.21	1.50	1.20	1.90	0.37	0.26	0.53	2.95	2.45	3.36
Median	*0.46*	*0.52*	*0.41*	*37.7*	*38.8*	*38.8*	*76.8*	*70.4*	*72.9*	*1.22*	*1.24*	*1.17*	*2.45*	*2.10*	*2.45*	*1.28*	*0.91*	*1.16*	*4.20*	*3.78*	*4.01*
**Group: Vit C + Flav**																					
**2** [a]	0.38	0.49	0.21	34.4	33.3	32.2	71.5	62.6	63.4	1.32	1.37	1.36	3.00	3.20	3.30	1.92	1.85	1.56	5.18	5.36	5.35
6	0.63	0.48	-	35.5	33.3	-	67.3	64.1	-	1.84	1.91	-	4.20	4.60	-	1.27	1.50	-	6.61	7.22	-
9	4.66	4.46	8.05	35.5	35.5	36.6	81.7	83.8	84.0	1.21	1.32	1.43	3.80	3.60	3.40	2.10	1.09	0.97	5.92	5.45	5.26
10	0.40	0.53	1.43	62.8	70.5	63.9	73.5	69.1	80.0	0.75	0.62	0.59	1.20	1.40	1.50	2.29	2.42	2.05	3.00	3.11	3.04
11	0.36	0.99	0.39	36.6	36.6	36.0	69.8	68.0	67.5	1.52	1.48	1.35	4.10	3.50	3.50	1.42	1.00	1.23	6.29	5.45	5.37
12	0.30	0.30	0.67	38.8	40.7	40.5	69.0	62.7	66.2	1.60	1.54	1.52	1.30	1.40	1.30	0.78	1.08	0.89	3.28	3.43	3.21
**13** [a]	0.25	0.82	0.31	36.6	37.3	36.8	109.5	98.0	100.8	1.13	1.06	1.08	3.40	3.10	3.00	1.04	3.03	2.31	4.98	5.48	5.11
Median	*0.38*	*0.53*	*0.53*	*36.6*	*36.6*	*36.7*	*71.5*	*68.0*	*73.8*	*1.32*	*1.37*	*1.36*	*3.40*	*3.20*	*3.15*	*1.42*	*1.50*	*1.40*	*5.18*	*5.45*	*5.19*

[a] Subject received supplements with only 500 mg of vitamin C (without flavonoids).
-, represents no data available, patient did not attend visit T2.

Table S4. Vitamin C levels at T0, T1 and T2.

	Subject	Vitamin C (mg/L)		
		**T0**	**T1**	**T2**
**Group: fibers**				
	1	10.28	8.54	8.17
	4	7.57	8.81	7.05
	7	0.15	0.47	0.28
	8	37.32	12.37	15.85
	Median	*8.92*	*8.68*	*7.61*
**Group: Vit C + Flav**				
	2[a]	7.03	19.58	17.00
	6	4.53	15.52	
	9	5.57	20.91	10.25
	10	6.97	13.14	7.81
	11	14.44	29.85	20.86
	12	19.78	20.28	21.82
	13 [a]	14.37	18.04	20.71
	Median	*7.03*	*19.58*	*18.86*

[a] Subject received supplements with only 500 mg of vitamin C (without flavonoids).

Table S5. Consumption of fruit, vegetables, and wine at T0, T1, T2.

**Subject**	**Fruit past week (nr. of units)**			**Fruit yesterday (nr. of units)**			**Vegetables past week (nr. of 100 gr.)**			**Vegetables yesterday (nr. of 100 gr.)**			**Wine past week (nr. of units)**			**Wine yesterday (nr. of units)**		
	**T0**	**T1**	**T2**	**T0**	**T1**	**T2**	**T0**	**T1**	**T2**	**T0**	**T1**	**T2**	**T0**	**T1**	**T2**	**T0**	**T1**	**T2**
**Group: Fibers**																		
1	0	7	6	0	1	0	7	8	5	1	2	1	1	2	2	1	0	0
4	3	6	7	0	0	1	7	2	7	1	0	0	14	3	4	2	0	0
7	0	0	2	0	0	1	1	3	4	1	0	0	0	0	0	0	0	0
8	4	7	7	0	1	2	7	7	7	1	0	0	0	1	2	0	0	0
Median	*1.5*	*6.5*	*6.5*	*0.0*	*0.5*	*1.0*	*7.0*	*4.8*	*6.0*	*1.0*	*0.0*	*0.0*	*0.5*	*1.5*	*2.0*	*0.5*	*0.0*	*0.0*
**Group: Vit C + Flav**																		
**2**[a]	7	7	7	1	2	1	11	11	4	1	0	0	0	3	2	0	0	2
6	7	5		1	2		5	5		1	1		0	0		0	0	
9	6	7	7	3	1	2	6	7	5	1	1	0	0	0	0	0	0	0
10	4	16	14	2	2	2	7	7	2	1	1	0	0	0	0	0	0	0
11	7	7	1	1	1	0	7	6	3	1	2	1	12	3	3	3	0	0
12	7	6	11	2	1	2	7	7	7	1	1	1	7	6	4	1	0	1
**13**[a]	14	7	7	2	1	0	7	7	10	1	1	2	0	0	0	0	0	0
Median	*7.0*	*7.0*	*7.0*	*2.0*	*1.0*	*1.5*	*7.0*	*7.0*	*4.5*	*1.0*	*1.0*	*0.5*	*0.0*	*0.0*	*1.0*	*0.0*	*0.0*	*0.0*

[a] Subject received supplements with only 500 mg of vitamin C (without flavonoids).
